# Analysis of survival-related factors in patients with endometrial cancer using a Bayesian network model

**DOI:** 10.1371/journal.pone.0314018

**Published:** 2024-11-21

**Authors:** Huan Zhang, Shan Zhao, Pengzhong Lv

**Affiliations:** Department of Obstetrics and Gynecology, The First Affiliated Hospital of Shandong First Medical University & Shandong Provincial Qianfoshan Hospital, Jinan, Shandong, P.R. China; Instituto do Cancer do Estado de Sao Paulo / University of Sao Paulo, BRAZIL

## Abstract

**Background:**

In recent years, remarkable progress has been made in the use of machine learning, especially in analyzing prognosis survival data. Traditional prediction models cannot identify interrelationships between factors, and the predictive accuracy is lower. This study aimed to construct Bayesian network models using the tree augmented naïve algorithm in comparison with the Cox proportional hazards model.

**Methods:**

A Bayesian network model and a Cox proportional hazards model were constructed to analyze the prognostic factors of endometrial cancer. In total, 618 original cases obtained from the Surveillance, Epidemiology, and End Results database were used to construct the Bayesian network model, which was compared with the traditional Cox proportional hazards model by analyzing prognostic factors. External validation was performed using a dataset from The First Affiliated Hospital of Shandong First Medical University.

**Results:**

The predictive accuracy, area under the receiver operating characteristic curve, and concordance index for the Bayesian network model were 74.68%, 0.787, and 0.72, respectively, compared to 68.83%, 0.723, and 0.71, respectively, for the Cox proportional hazards model. Tumor size was the most important factor for predicting survival, followed by lymph node metastasis, distant metastasis, chemotherapy, lymph node resection, tumor stage, depth of invasion, tumor grade, histological type, age, primary tumor site, radiotherapy and surgical sequence, and radiotherapy.

**Conclusion:**

The findings indicate that the Bayesian network model is preferable to the Cox proportional hazards model for predicting survival in patients with endometrial cancer.

## Introduction

Endometrial cancer (EC) is a common cancer in women worldwide and one of the leading causes of cancer death in the United States. An estimated 66,200 new cases of cancer of the uterine corpus and almost 13,030 deaths attributable to this disease are projected to occur in 2023 [1]. At present, surgery and radiotherapy are the main treatment methods for EC. However, many associated factors affect outcomes. Studies identified age, menopause, tumor size, depth of myometrial infiltration [2], lymphatic metastasis, pathological type, histological grading, cervical involvement, marital status [3], and lymph vascular infiltration [4] as prognostic factors [5]. The International Federation of Gynecology and Obstetrics (FIGO) staging system has been used to estimate prognosis, but it has low predictive accuracy [6]. Thus, various risk stratification models have been proposed to predict survival. It is recognized that the accurate prediction of outcomes depends on a complex and dynamic interplay of multiple variables that might be inadequately captured by traditional multivariate statistical modeling. The Cox proportional hazards model (CPH) has been widely used in survival analysis. For example, Niu et al. established a prognosis prediction model for esophageal cancer based on autophagy-related genes using the Cox proportional hazards model [7]. Additionally, Ouyang et al. used the Cox proportional hazards model to establish a survival prediction model for patients with lung cancer [8]. However, the accuracy of the Cox proportional hazards model is unsatisfactory because of its low predictive accuracy.

A Bayesian network is a probabilistic graphical model that represents a set of variables and their conditional dependencies through a directed acyclic graph [9]. The causal relationship can be visualized [10]. The directed acyclic graph of the Bayesian network contains nodes and arrows, with nodes representing variables and arrows representing the dependence in probability between the parent and child variables [11]. Some studies found that Bayesian neural networks were preferable to artificial neural networks for survival prediction and classification [12–14]. Furthermore, when compared to the traditional Cox proportional hazards model, the Bayesian network showed better performance in predicting survival. For example, the Bayesian network demonstrated superior accuracy compared to the Cox regression-based nomogram in predicting survival time among gallbladder carcinoma patients who underwent curative-intent resection [15]. Additionally, the Bayesian network outperformed the Cox proportional hazard model in individual-year predictions of patient survivorship in breast cancer cases [16]. Bayesian network models have been applied to predict lymph node metastasis in endometrial carcinoma [17, 18].

In the present study, we used the Surveillance, Epidemiology, and End Results (SEER) database to construct a prognostic prediction model of EC using a Bayesian network to explore the prognostic factors of EC. Furthermore, we compared the accuracy of the Bayesian network model with that of the Cox proportional hazards model.

## Materials and methods

### Patients

The SEER database is the authoritative cancer statistical database in the United States, containing clinical information for nearly 34.6% of the American population with malignant tumors [19]. The records of 618 patients in the SEER database who were diagnosed with EC between 2010 and 2015 were obtained using SEER Stat software (version 8.3.9). These data are available for everybody upon request and are part of a standard statistical report.

Meanwhile, 104 patients with EC treated at The First Affiliated Hospital of Shandong First Medical University from 2010 to 2016 were selected for external validation. The authors had access to information that could identify individual participants during data collection from 104 patients. This study was approved by the Medical Ethics Committee of The First Affiliated Hospital (Qianfoshan Hospital), Shandong First Medical University (No.**YXLL-KY-2023(030)**).

The primary objective of this study is to predict the five-year survival status of patients with EC. Accordingly, the follow-up period is set to a duration of up to five years.

Patients who met the following criteria were included in the study: (i) radical resection was performed; (ii) diagnosis of primary endometrial malignancy; and (iii) their records contained complete clinicopathological features and follow-up data. The exclusion criteria were as follows: (i) a history of malignant tumors or coincidence of other malignant tumors; and (ii) death of non-EC–related disease. The following variables were collected: age, tumor grade, histological type, tumor stage, radiotherapy and surgical sequence, radiotherapy, chemotherapy, lymph node resection, lymph node metastasis, tumor size, depth of invasion, distant metastasis, and survival time. The dataset was randomly divided into a training dataset (75%, *n* = 464) and a testing dataset (25%, *n* = 154) using the “rand” function in Microsoft Excel.

### Statistical analysis

The calculations involved in this study were implemented using R 4.0.1.

The Cox proportional hazards model for survival analysis is a semi-parametric regression model proposed by Cox that has been widely used in medicine, sociology, and other fields because of its superiority in handling censored data.

The Cox proportional hazards model is designed to simultaneously evaluate the effect of several factors on survival. Let *X* = (*X*_1_,*X*_2_,…,*X*_*P*_) denote the covariates affecting survival status. The Cox model is then expressed by the hazard function denoted by *h*(*t*,*X*), which can be interpreted as the risk of dying at time t. The hazard function can be estimated as follows:

$$h(t,X)=h_{0}(t)exp\{\beta^{T}X\},$$

where *t* represents the survival time, *h*(*t*,*X*) is the hazard function determined by a set of p covariates *X*_1_,*X*_2_,…,*X*_*P*_, the coefficients *β*_1_,*β*_2_,…,*β*_*P*_ measure the impact (i.e., the effect size) of covariates, and *h*_0_(*T*) is the baseline hazard, corresponding to the value of the hazard when all covariates *X*_*i*_ are equal to zero.

A Bayesian network is a directed acyclic graph containing nodes representing variables and arrows indicating the dependence in probability between parent and child nodes. Friedman et al. [20] proposed a tree-augmented plain Bayesian classifier that relaxes limitations on conditional independence, allowing more complex and realistic relationships among different variables to be modeled. The diagram expressing the dependencies among attributes is presented as a tree structure.

The tree augmented naïve algorithm consists of four steps: (i) calculate the mutual information function between different attribute pairs; (ii) establish an undirected graph; (iii) build a maximum weight-spanning tree; and (iv) select the root node and set the edges that point from the root to the other nodes, converting an undirected tree to a directed acyclic tree.

### Evaluation index

Confusion matrices are extremely popular measures used in solving classification problems. Confusion matrices represent counts from predicted and actual values. The output true negative (TN) indicates the number of negative examples classified accurately. Similarly, true positive (TP) indicates the number of positive examples classified accurately. The term false positive (FP) denotes the number of actual negative examples incorrectly classified as positive, and false negative (FN) represents the number of actual positive examples incorrectly classified as negative. One of the most commonly used metrics in performing classification is accuracy. The accuracy of a model (through a confusion matrix) is calculated using the following formula:

$$Accuracy=\frac{TP+TN}{TP+FP+TN+FN}$$

However, when the number of negative and positive cases varies greatly, the accuracy index might not be an appropriate evaluation index. With this in mind, we plotted the receiver operating characteristic (ROC) curve and calculated the area under the ROC curve (AUC) to measure the overall performance of the classification model.

At the same time, the concordance index (C-index) has emerged as a prevalent alternative to traditional metrics such as accuracy and AUC. The C-index is extensively utilized for the comprehensive evaluation of prognostic models in survival analysis. Similar to the AUC, the C-index provides a summary of model performance across various possible configurations. Its distinguishing feature, however, is its ability to characterize a model’s prognostic capability by accounting for both outcome occurrence and timing [21].

## Results

### Characteristics of patients with EC

In total, 618 postoperative patients were included in this study. Of these, 192 patients died, whereas 426 survived for at least 5 years. Recall that our follow-up period is set to a maximum duration of five years, with the maximum follow-up length being 60 months and the minimum follow-up length being 1 month. The mean follow-up length was 50.01 months, and the median was 60 months. Detailed clinicopathological characteristics of the patients are presented in S1 Table of the Supporting information.

### Univariate and multivariate Cox regression analyses

The Cox proportional hazards model was constructed based on 464 patients in the training dataset. Unilateral Cox regression screening variables were used to conduct univariate analysis. Factors associated with the prognosis of patients with EC were identified as follows: patient age, tumor grade, histological type, tumor stage, chemotherapy, lymph node resection, lymph node metastasis, tumor size, depth of invasion, and distant metastasis (Table 1).

**Table 1:** Univariate Cox regression analysis.

Variable	LR	df	P
**Age**	39.88	4	<0.001
**Tumor site**	0.21	1	0.644
**Tumor grade**	40.13	3	<0.001
**Histological type**	35.45	5	<0.001
**Tumor stage**	59.25	3	<0.001
**Radiotherapy and surgical sequence**	2.31	2	0.315
**Radiotherapy**	0.68	1	0.411
**Chemotherapy**	7.92	1	0.005
**Lymph node resection**	10.36	1	0.001
**Lymph node metastasis**	15.03	1	<0.001
**Tumor size**	12.33	1	<0.001
**Depth of invasion**	26.65	2	<0.001
**Distant metastasis**	22.88	1	<0.001

**Abbreviations: LR:** Likelihood Ratio **df:** Degrees of Freedom **P**: P-value

Cox regression was used for a multifactor analysis to determine which univariate prognostic relationships were independent predictors. The results suggested that age, tumor grade, tumor stage, chemotherapy, and tumor size are independent risk factors affecting EC prognosis (*P*<0.05, Table 2).

**Table 2:** Multivariate Cox regression analysis.

		coef	exp(coef)	se(coef)	z	Pr(>|z|)
**Age**	<50	0	1			
	50–59	0.210	1.234	0.369	0.570	0.568
	60–69	0.787	2.198	0.351	2.244	0.025
	70–79	1.064	2.898	0.367	2.900	0.004
	>80	1.456	4.289	0.422	3.452	<0.001
**Tumor grade**	High differentiation	0	1			
	Middle differentiation	1.327	3.77	0.530	2.510	0.012
	Low differentiation	1.645	5.180	0.526	3.125	0.002
	Undifferentiation	1.770	5.870	0.568	3.114	0.002
**Histological type**	Endometrioid carcinoma	0	1			
	Serous carcinoma	0.303	1.354	0.247	1.226	0.220
	Clear cell carcinoma	0.682	1.978	0.447	1.527	0.127
	Undifferentiated carcinoma	0.558	1.747	0.809	0.690	0.490
	Mixed cell carcinoma	-0.264	0.76	0.313	-0.844	0.399
**Tumor stage**	I	0	1			
	II	0.182	1.200	0.397	0.459	0.646
	III	0.994	2.702	0.312	3.188	0.001
	IV	1.267	3.550	0.413	3.068	0.002
**Radiotherapy**	No	0	1			
	Yes	-0.471	0.624	0.236	-1.994	0.046
**Lymph node resection**	No	0	1			
	Yes	-0.351	0.704	0.259	-1.353	0.176
**Lymph node metastasis**	No	0	1			
	Yes	0.193	1.213	0.232	0.834	0.404
**Tumor size**	<4cm	0	1			
	≥4cm	0.366	1.443	0.1780	2.058	0.040
**Depth of invasion**	Confined endometrial layer	0	1			
	<1/2 Muscular layer	0.435	1.544	1.0533	0.413	0.680
	≥1/2 Muscular layer	1.309	3.703	1.0209	1.282	0.200
**Distant metastasis**	No	0	1			
	Yes	0.293	1.341	0.3691	0.795	0.427

**Abbreviations: coef**: Coefficient **exp(coef):** Exponentiated Coefficient (Hazard Ratio)

**se(coef):** Standard Error of Coefficient z: Z-statistic **Pr(>|z|):** P-value

### Constructing a Cox proportional hazards model

The 5-year survival status of the 154 patients in the test set was predicted using a multifactorial Cox proportional hazards model. The reliability and accuracy of the predictions were determined using confusion matrices. The probability threshold was calculated using Jorden’s rule. The data revealed that 75 (>5 years) and 31 patients (≤5 years) were correctly classified, giving a model accuracy of 68.83%, as presented in Table 3.

**Table 3:** Confusion matrix (CPH).

		True value		Prediction accuracy
		Alive	Dead	
**Predicted value**	**Alive**	75	17	68.83%
	**Dead**	31	31	

**Fig 1:**
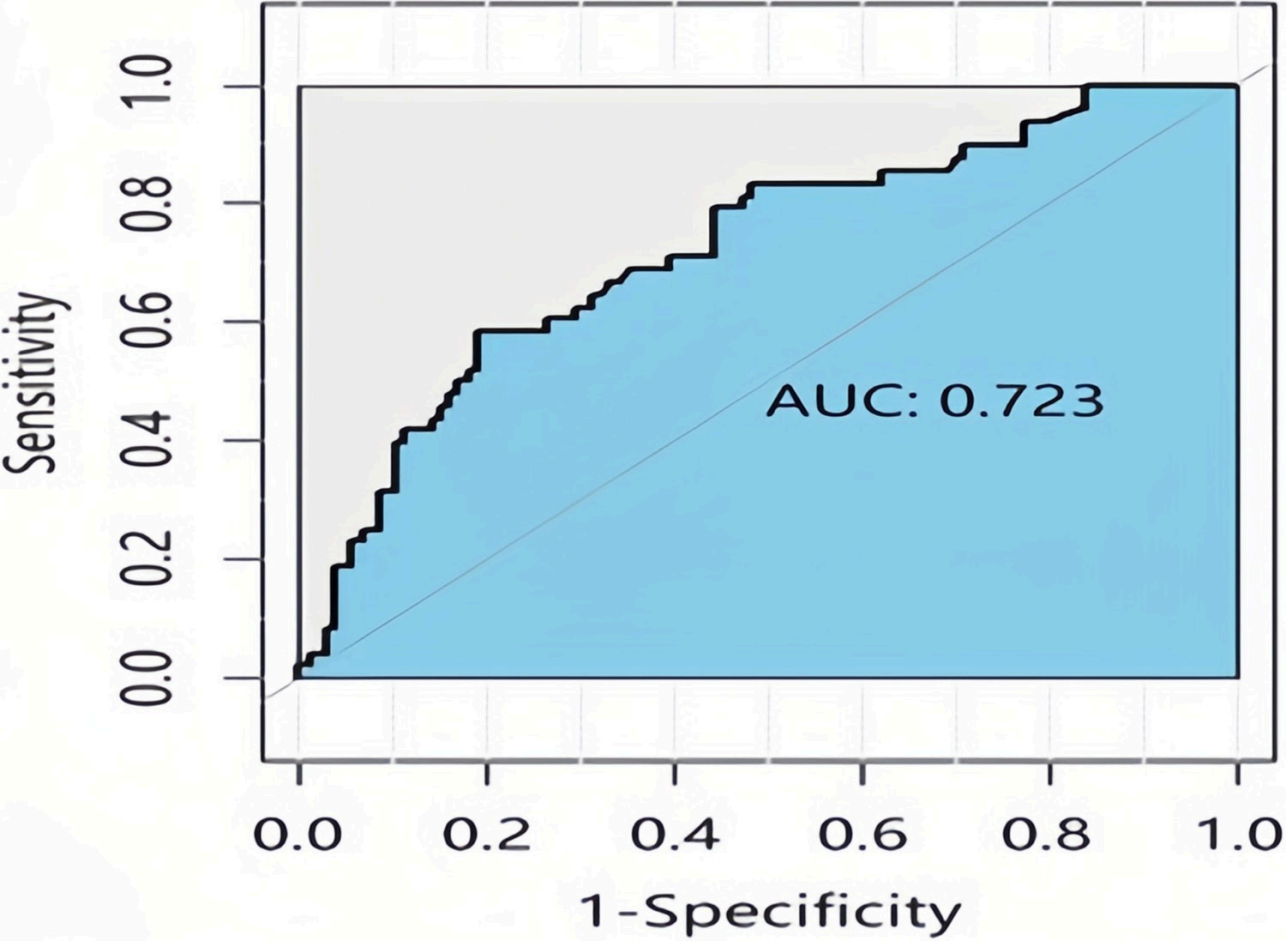
ROC curve for validation of the Cox proportional hazard model. The AUC of the Cox proportional hazards model was 0.723. AUC values range from 0 to 1, with values closer to 1 indicating better classifier performance.

The AUC of the Cox proportional hazards model was 0.723, as shown in Fig 1. Additionally, the C-index value of the model was 0.71.

### Bayesian network model construction

**Fig 2:**
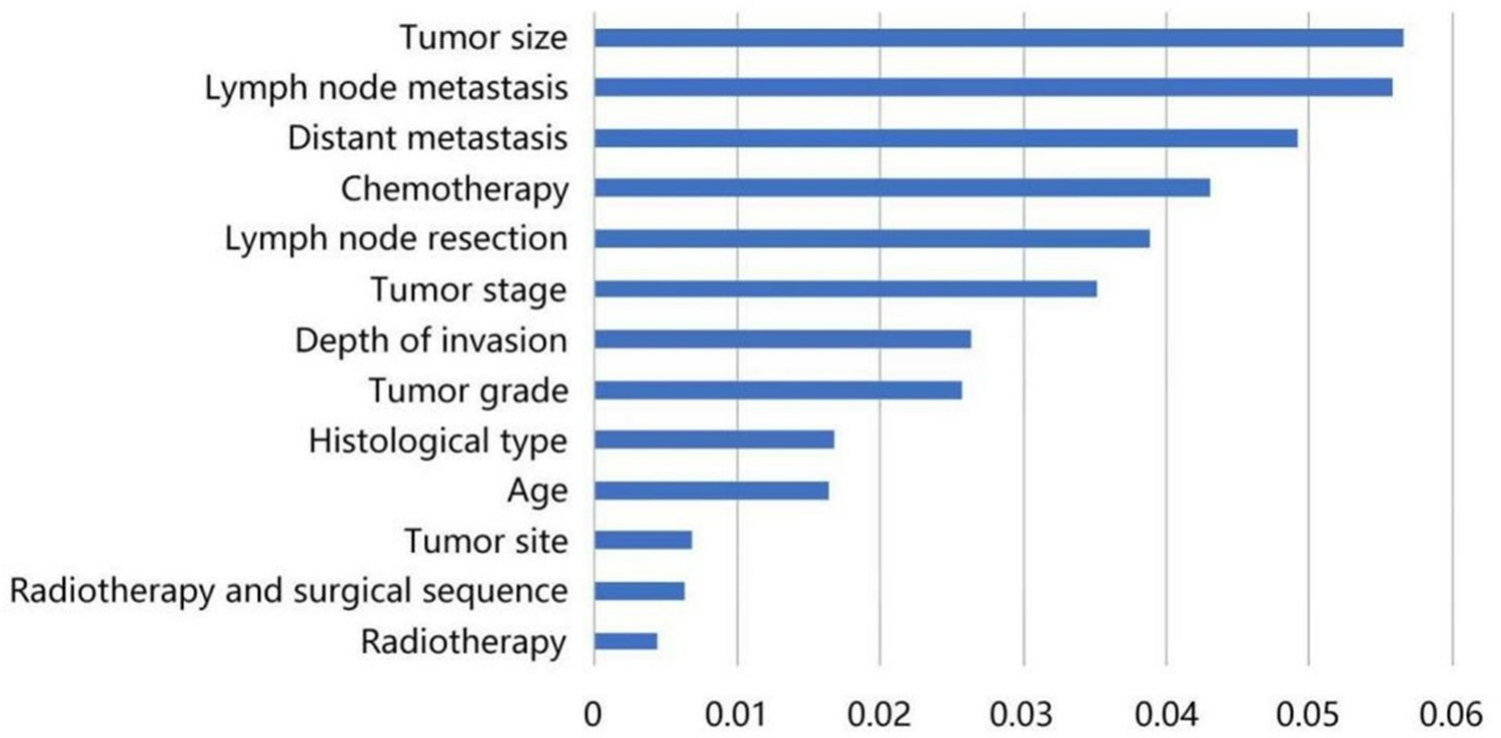
Independent variable impact strength ranking. These factors were ordered by their MMFV values as follows: tumor size, lymph node metastasis, distant metastasis, chemotherapy, lymph node resection, tumor stage, depth of invasion, tumor grade, histological type, age, primary tumor site, radiotherapy and surgical sequence, and radiotherapy.

**Fig 3:**
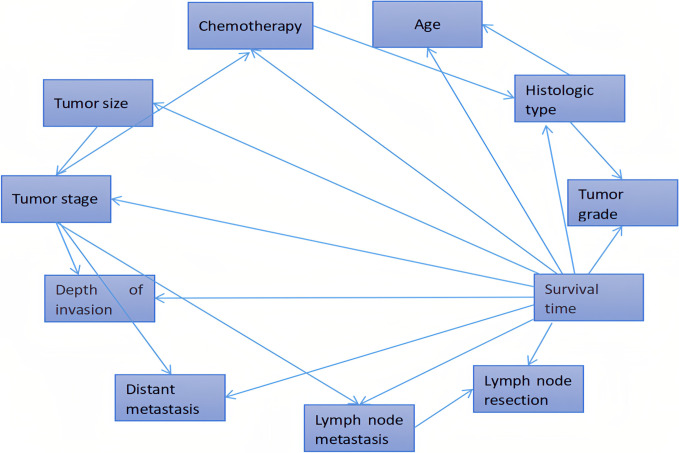
Bayesian network model for prognostic factors. The arrow is directional and represents cause-and-effect relationships as well as associations.

First, we calculated the probability distribution of patients at different levels for each variable and described it in the following form: {P(V=p),(p=1),⋯}. Next, the posterior probability distribution of patient survival at 5 years was calculated for each level, as denoted by {P(Alive|V=0,P(Alive|V=1),⋯}. Finally, the importance measure mean multi-state Fussell–Vesely (MMFV) was calculated for each variable by combining the prior probability that the patient would survive for 5 years. The results are presented in Table 4. Moreover, the independent influencing factors were ranked according to their MMFV values, as presented in Fig 2. According to the MMFV of each variable (influencing factor), each variable was added to the training model in turn. To address the issues of overfitting and underfitting, the Bayesian network introduces 10 variables, including tumor size, lymph node metastasis, distant metastasis, chemotherapy, lymph node resection, tumor stage, depth of invasion, tumor grade, histologic type, and age. The construction of the Bayesian network is presented in Fig 3.

**Table 4:** Variable importance measure.

Variable	Level	P(V = j)	P(Alive|V = j)	MMFV	rank
**Age**	**<**50	0.138	0.797	0.016	10
	50–59	0.293	0.794		
	60–69	0.336	0.654		
	70–79	0.172	0.613		
	>80	0.061	0.357		
**Tumor site**	Fundus of the uterus	0.019	0.444	0.007	11
	Rest of uterine cavity	0.98	0.695		
**Tumor grade**	High differentiation	0.120	0.933	0.025	8
	Middle differentiation	0.334	0.755		
	Low differentiation	0.420	0.626		
	Undifferentiation	0.116	0.463		
**Histological type**	Endometrioid carcinoma	0.675	0.786	0.017	9
	Serous carcinoma	0.170	0.494		
	Clear cell carcinoma	0.023	0.455		
	Undifferentiated carcinoma	0.011	0.600		
	Mixed cell carcinoma	0.121	0.625		
**Tumor stage**	I	0.342	0.874	0.035	6
	II	0.121	0.768		
	III	0.356	0.624		
	IV	0.181	0.417		
**Radiotherapy and surgical sequence**	Operation only	0.088	0.610	0.006	12
	Postoperative radiotherapy	0.903	0.699		
	Preoperative radiotherapy	0.009	0.500		
**Radiotherapy**	No	0.088	0.818	0.004	13
	Yes	0.912	0.687		
**Chemotherapy**	No	0.457	0.755	0.043	4
	Yes	0.543	0.635		
**Lymph node resection**	No	0.136	0.049	0.039	5
	Yes	0.864	0.721		
**Lymph node metastasis**	No	0.763	0.740	0.056	2
	Yes	0.237	0.527		
**Tumor size**	<4cm	0.584	0.756	0.057	1
	≥4cm	0.416	0.596		
**Depth of invasion**	Confined endometrial layer	0.037	0.882	0.026	7
	<1/2 Muscular layer	0.192	0.843		
	≥1/2 Muscular layer	0.771	0.643		
**Distant metastasis**	No	0.867	0.739	0.049	3
	Yes	0.133	0.446		

**Abbreviation: MMFV**: Measure Mean Multi-state Fussell–Vesely

**Fig 4:**
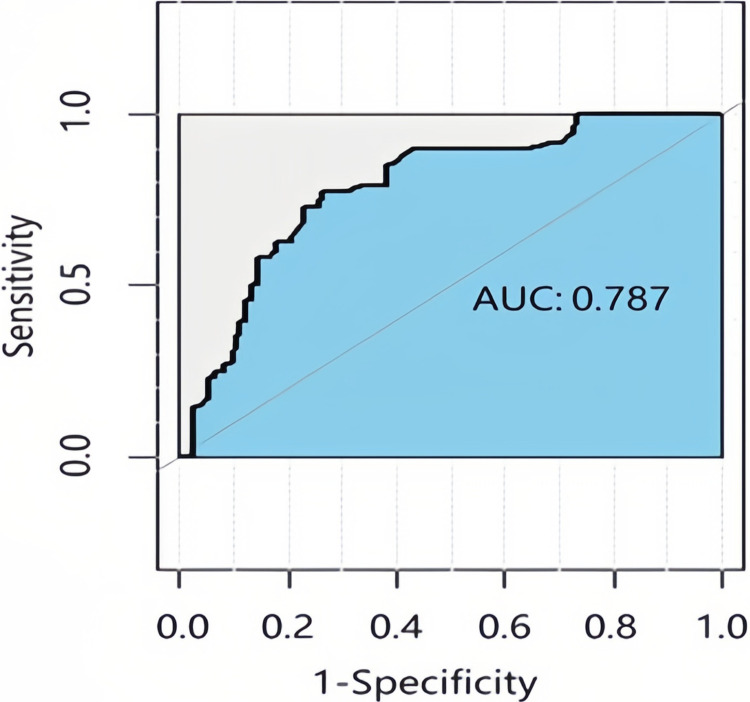
ROC curve for validation of the Bayesian network model. The AUC of the Bayesian network model was 0.787. AUC values range from 0 to 1, with values closer to 1 indicating better classifier performance.

The constructed Bayesian network was used to predict the 5-year survival of 154 patients in the test set. The reliability and accuracy of the predictions were obtained using a confusion matrix. The data revealed that the survival of 79 (>5 years) and 36 patients (≤5 years) was correctly classified, giving a model accuracy of 74.68%, as presented in Table 5. The AUC of the Bayesian network model was 0.787, as shown in Fig 4. The C-index value of the Bayesian network model was 0.72.

**Table 5:** Confusion matrix (BN).

		True value		Prediction accuracy
		Alive	Dead	
**Predicted value**	**Alive**	79	12	74.68%
	**Dead**	27	36	

To compare the performance of the CPH model and the BN model, we employed the following metrics: the C-Index, which assesses the accuracy of predictions regarding patient mortality risk, and the Accuracy and AUC, which evaluate the models’ ability to predict patients’ future survival status. These results are summarized in Table 6.

**Table 6:** Performance metrics.

Model	C-Index	Accuracy	AUC
**CPH**	0.71	68.83%	0.723
**BN**	0.72	74.68%	0.787

**Abbreviations: AUC:** Area Under the Curve **CPH:** Cox Proportional Hazards Model

**BN:** Bayesian Network

As illustrated in Table 6, the Bayesian network model exhibited superior performance compared to the Cox model in the SEER cohort.

### External validation of the Bayesian network models

The dataset from patients with EC who underwent surgery between 2010 and 2016 at our hospital was obtained. A total of 104 patients were successfully recruited. The maximum follow-up length was 60 months, and the minimum follow-up length was 5 months, the median is 60 months, the mean follow-up length was 56.82 months. The clinicopathological characteristics of these patients are detailed in S2 Table of the Supporting information.

The aforementioned Bayesian network model was externally validated using 104 clinical patient cases. The reliability and accuracy of the predictions were obtained using confusion matrix assessment indicators. The survival of 47 (>5 years) and 24 patients (≤5 years) was correctly classified, giving a model accuracy of 68.27%, as presented in Table 7.

**Table 7:** Confusion matrix of external validation (BN).

		True value		Prediction accuracy
		Alive	Dead	
**Predicted value**	**Alive**	47	0	68.27%
	**Dead**	33	24	

**Fig 5:**
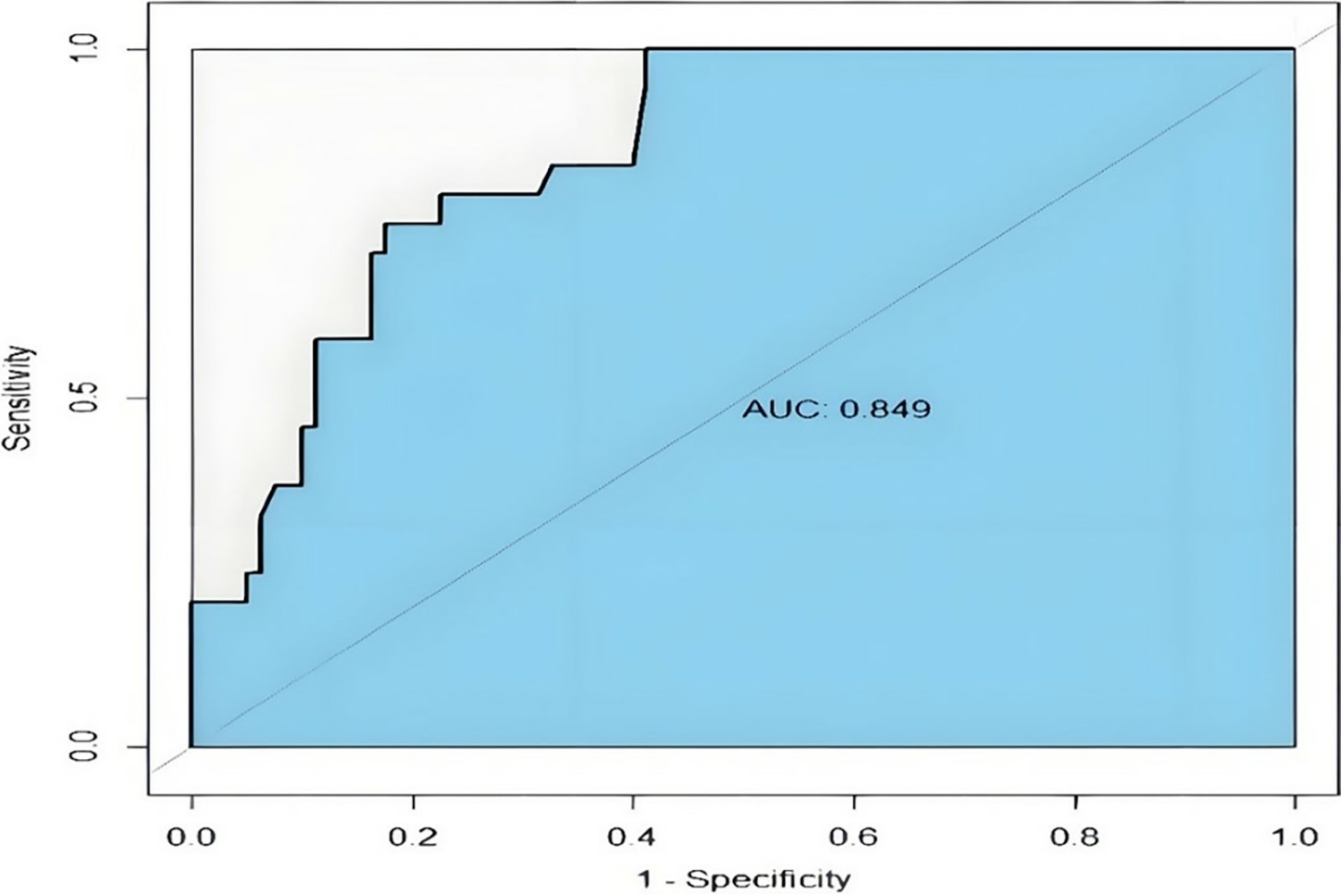
ROC curve for external validation of the Bayesian network model. The AUC of the Bayesian network model was 0.849. Estimates of AUC values range from 0 to 1, with values closer to 1 indicating better performance of the classifier.

The AUC of the Bayesian network model was 0.849, as presented in Fig 5.

**Fig 6:**
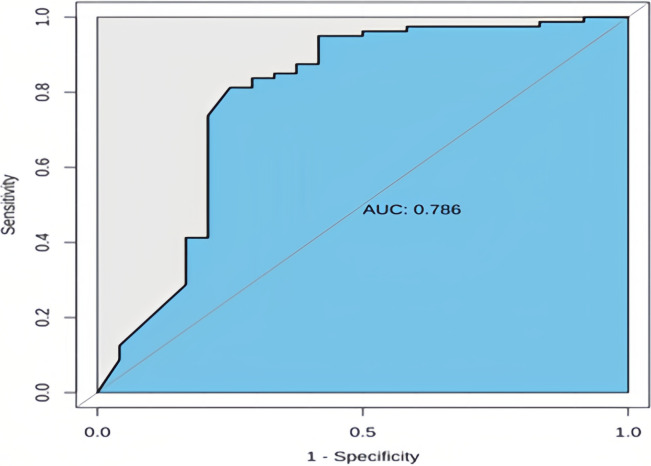
ROC curve for external validation of the Cox proportional hazards model. The CPH model achieved an AUC of 0.786. AUC values range from 0 to 1, with scores closer to 1 indicating better classifier performance.

To evaluate the performance of the Bayesian network model on external data, we also employed a previously established Cox proportional hazards model for prediction. The corresponding Area Under the Curve (AUC) for this model was 0.786, as illustrated in Fig 6, with an accuracy of 66.35% (see Table 7).

Furthermore, we calculated the C-index values for external validation of both the Bayesian network (BN) and Cox proportional hazards (CPH) models. These results are presented in Table 8 below.

**Table 8:** Performance metrics.

Model	C-Index	Accuracy	AUC
**CPH**	0.79	66.35%	0.786
**BN**	0.81	68.27%	0.849

**Abbreviations**: **C-Index**: Concordance Index **AUC**: Area Under the Curve

**CPH**: Cox Proportional Hazards Model **BN**: Bayesian Network

As demonstrated in Table 8, the Bayesian network model displayed better predictive performance in the external validation cohort, outperforming the Cox proportional hazards model in terms of accuracy, AUC, and C-index.

## Discussion

EC is the fourth most common malignant disorder in the United States. An accurate and reliable prediction model for postoperative patients will facilitate assessment of the prognostic status and allow the creation of appropriate treatment plans.

To analyze the factors related to patient survival, the Cox proportional hazards model has been widely used in cancer patient survival prediction. It assumes that the risk rate of an individual with a given set of risk factors is a multiple of the unspecified baseline risk function and the regression component [22]. In this study, the Cox proportional hazards model suggested that age, tumor grade, tumor stage, and tumor size were independent risk factors affecting the outcomes of patients with EC (all P<0.05). These findings are consistent with those of previous studies [5, 23–25]. Advanced age is associated with more adverse disease features, adjuvant treatment, and poorer outcomes. we found that chemotherapy serves as an independent prognostic factor for patients with EC after surgery. However, lymph node metastasis was not identified as an independent risk factor, while stage III emerged as an independent risk factor for patient survival in multifactorial analysis. This was mainly because this model did not consider the relationship between stage III and lymph node metastasis. Therefore, the Cox proportional hazards model overlooks the interaction among prognostic factors and their combined effect on cancer prognosis. It is often less accurate in predicting patient survival, and its accuracy was only 68.83% in our study. Thus, a new modeling method is required to overcome this deficiency.

Machine learning, a significant branch of artificial intelligence, has been widely applied in the medical field [26–29]. The Bayesian network, a classical algorithm in machine learning, offers the advantages of a rigorous probabilistic framework for inferring multiple variables, along with an interactive and easily interpretable visual representation. Bayesian networks have demonstrated superior predictive performance compared to traditional statistical methods, enabling their application across various domains. For example, in environmental engineering practice, the Bayesian network model has been used to address the overfitting problem of different variables in the calibration process [30]. In soil properties, the Bayesian model is an important tool across the ecological scale of land use types [31]. In the medical field, it has been widely used for diverse applications, including estimating disease incidence and predicting survival [32, 33].

In the present study, we constructed a Bayesian network model using data from patients with EC in the SEER database to predict prognosis, followed by external validation. The correlations of predictive factors were obtained using the Bayesian network model. Moreover, these factors were ordered by their MMFV values as follows: tumor size, lymph node metastasis, distant metastasis, chemotherapy, lymph node resection, tumor stage, depth of invasion, tumor grade, histological type, age, primary tumor site, radiotherapy and surgical sequence, and radiotherapy. The Bayesian network model correctly classified 79 patients with survival times of >5 years and 36 patients with survival times of ≤5 years, giving a model accuracy of 74.68%. The AUC and C-index of the Bayesian network model were 0.787 and 0.72, respectively. Conversely, the Cox proportional hazards model correctly classified 76 patients with survival times of >5 years and 29 patients with survival times of ≤5 years, with an accuracy of 68.83%. Furthermore, the AUC and C-index of the Cox proportional hazards model were 0.723 and 0.71, respectively, demonstrating that the Bayesian network model outperformed the Cox proportional hazards model.

We also utilized a dataset of 104 patients with EC from our hospital for external validation of the Bayesian network model. The corresponding AUC and C-index were 0.849 and 0.81, respectively. These external validation results confirmed the high predictive accuracy of the Bayesian network model. In addition, the Bayesian network model reflects the relationship between variables. In our study, we found that histological type was correlated with age at diagnosis, tumor grade, and chemotherapy. However, in our external validation, the accuracy was only 68.27%, which we attribute primarily to the limited sample size of the study. Therefore, a larger dataset is needed to construct a Bayesian model with higher accuracy.

While the Bayesian networks demonstrate superior predictive performance compared to the Cox proportional hazards model, the Cox proportional hazards model provides better interpretability regarding the effects of specific variables on patient prognosis. This interpretability helps strengthen shared decision-making between patients and clinicians. Therefore, integrating the Bayesian network and Cox proportional hazards model can provide more explanatory and accurate adjuvant therapy planning for the individualized evaluation of patients with EC. In addition to the accuracy provided by Bayesian networks, the explainable treatment options are sufficient to give patients confidence in the recommended treatment.

This study confirmed the high predictive accuracy of the Bayesian network model, and the validation results suggest its potential for widespread use in clinical research. However, several limitations should be considered. A significant number of samples were excluded due to inadequate variable information. Some clinicopathological features were also not included, such as menopause, vaginal bleeding, and lymph vascular interstitial infiltration. Therefore, further improvement of our model is necessary. Integrating Bayesian networks with Cox proportional risk models can greatly improve the accurate estimation of prognostic influences and overall survival in EC, as well as enhance patient management-related decisions. For patients with EC, numerous prognostic factors affecting their survival should be evaluated to develop individualized treatment plans to improve patient survival rates.

## Conclusion

The Bayesian network model displayed great promise as a reliable and accurate risk stratification tool for clinical decision-making. This is due to its ability to account for dynamic, nonlinear interactions between clinical and nonclinical variables, as well as their interdependencies in influencing outcomes. We analyzed clinicopathological factors influencing the prognosis of patients with EC after radical resection and constructed a five-year survival prediction Bayesian network model for the first time, which performed better than the Cox proportional hazards model in predicting the five-year survival status of patients with EC. The Bayesian network model constructed in this study can be used to guide the individualized treatment of patients with EC and provide an accurate prognostic evaluation tool for clinicians.

## Supporting information

S1 Table Clinicopathological characteristics of the entire cohort from SEER database. (DOCX)

S2 Table Clinicopathological characteristics of the entire cohort from the First Affiliated Hospital of Shandong First Medical University. (DOCX)
