# Supplementary material for: Analysis of survival-related factors in patients with endometrial cancer using a Bayesian network model
Source: PLoS One. 2024 Nov 21;19(11):e0314018. doi: 10.1371/journal.pone.0314018 (PMC11581279; doi:10.1371/journal.pone.0314018)
Supplement: S1 Table — (DOCX) [file pone.0314018.s001.docx]

**S1 Table.** **Clinicopathological characteristics of the entire cohort from SEER database**

| **Clinicopathological characteristics** | | **N （%）** | |
| --- | --- | --- | --- |
| **Age** | ＜50 | 85 | 13.754 |
|  | 50-59 | 192 | 31.068 |
|  | 60-69 | 201 | 32.524 |
|  | 70-79 | 107 | 17.314 |
|  | ＞80 | 33 | 5.340 |
| **Tumor site** | Fundus of the uterus | 10 | 1.618 |
|  | Rest of uterine cavity | 608 | 98.382 |
| **Tumor grade** | High differentiation | 91 | 14.725 |
|  | Middle differentiation | 198 | 32.039 |
|  | Low differentiation | 251 | 40.615 |
|  | Undifferentiation | 78 | 12.621 |
| **Histological type** | Endometrioid carcinoma | 421 | 68.123 |
|  | Mixed cell carcinoma | 78 | 12.621 |
|  | Serous carcinoma | 95 | 15.372 |
|  | Clear cell carcinoma | 18 | 2.913 |
|  | Undifferentiated carcinoma | 6 | 0.971 |
| **Tumor stage** | I | 208 | 33.657 |
|  | II | 72 | 11.650 |
|  | III | 215 | 34.790 |
|  | IV | 123 | 19.903 |
| **Radiotherapy and surgical sequence**  **radiotherapy and surgical sequence** | Operation only  Operation only | 51  51 | 8.252  8.3 |
|  | Postoperative radiotherapy | 560 | 90.615 |
|  | Preoperative radiotherapy | 7 | 1.133 |
| **Chemotherapy** | No | 280 | 45.307 |
|  | Yes | 338 | 54.693 |
| **Radiotherapy** | No | 51 | 8.252 |
|  | Yes | 567 | 91.748 |
| **Lymph node resection** | No | 85 | 13.754 |
|  | Yes | 533 | 86.246 |
| **Lymph node metastasis** | No | 464 | 75.081 |
|  | Yes | 154 | 24.919 |
| **Tumor size** | ＜４cm | 351 | 56.796 |
|  | ≥4cm | 267 | 43.204 |
| **Depth of invasion** | Confined endometrial layer | 23 | 3.722 |
|  | ＜1/2 Muscular layer | 117 | 18.932 |
|  | ≥1/2 Muscular layer | 478 | 77.346 |
| **Distant metastasis** | No | 524 | 84.790 |
|  | Yes | 94 | 15.210 |
