# Supplementary material for: Analysis of survival-related factors in patients with endometrial cancer using a Bayesian network model
Source: PLoS One. 2024 Nov 21;19(11):e0314018. doi: 10.1371/journal.pone.0314018 (PMC11581279; doi:10.1371/journal.pone.0314018)
Supplement: S2 Table — (DOCX) [file pone.0314018.s002.docx]

**S2 Table. Clinicopathological characteristics of the entire cohort from the First Affiliated Hospital of Shandong First Medical University**

| **Clinicopathological characteristics** | | **N （%）** | |  |
| --- | --- | --- | --- | --- |
| **Age** | ＜50 | 14 | 13.462 | |
|  | 50-59 | 53 | 50.961 | |
|  | 60-69 | 32 | 30.769 | |
|  | 70-79 | 5 | 4.808 | |
|  | ＞80 | 0 | 0 | |
| **Surgical methods** | Non-primary surgical procedure to distant site | 7 | 6.731 | |
|  | Any combo of sur proc to oth rg, dis lym nd, and/or dis site | 96 | 92.308 | |
|  | Non-primary surgical procedure performed | 1 | 0.961 | |
| **Tumor grade** | High differentiation | 42 | 40.385 | |
|  | Middle differentiation | 46 | 44.231 | |
|  | Low differentiation | 12 | 11.538 | |
|  | Undifferentiation | 4 | 3.846 | |
| **Tumor stage** | I | 65 | 62.500 | |
|  | II | 15 | 14.423 | |
|  | III | 20 | 19.231 | |
|  | IV | 4 | 3.846 | |
| **Radiotherapy and surgical sequence** | No radiation and/or cancer-directed surgery | 5 | 4.808 | |
|  | Radiation after surgery | 99 | 95.192 | |
| **Chemotherapy** | No | 56 | 53.846 | |
|  | Yes | 48 | 46.154 | |
| **Marital status** | Married (including common law） | 72 | 69.231 | |
|  | Widowed | 18 | 17.308 | |
|  | Divorced | 8 | 7.692 | |
|  | Separated | 5 | 4.808 | |
|  | Single (never married) | 1 | 0.961 | |
| **Lymph node metastasis** | No | 81 | 77.885 | |
|  | Yes | 23 | 22.115 | |
| **Depth of invasion** | Confined endometrial layer | 47 | 45.192 | |
|  | ＜1/2 Muscular layer | 42 | 40.385 | |
|  | ≥1/2 Muscular layer | 15 | 14.423 | |
| **Distant metastasis** | No | 88 | 84.615 | |
|  | Yes | 16 | 15.385 | |
